# Home Dampness Signs in Association with Asthma and Allergic Diseases in 4618 Preschool Children in Urumqi, China-The Influence of Ventilation/Cleaning Habits

**DOI:** 10.1371/journal.pone.0134359

**Published:** 2015-07-31

**Authors:** Zhijing Lin, Zhuohui Zhao, Huihui Xu, Xin Zhang, Tingting Wang, Haidong Kan, Dan Norback

**Affiliations:** 1 Department of Environmental Health, School of Public Health, Key Laboratory of Public Health Safety, Ministry of Education, Fudan University, Shanghai, 200032, P. R. China; 2 Department of Environmental Health, Shanghai Municipal Center for Disease Control and Prevention, Shanghai, 200032, China; 3 Institute of Environmental Science, Shanxi University, Taiyuan, 030006, China; 4 Department of Maternal, Child and Adolescent Health, School of Public Health, Xinjiang Medical University, Urumqi, China; 5 Department of Medical Sciences, Occupational and Environmental Medicine, Uppsala University, Uppsala, SE-751, Sweden; Peking University, CHINA

## Abstract

There is an increasing prevalence of childhood asthma and allergic diseases in mainland of China. Few studies investigated the indoor dampness, ventilation and cleaning habits and their interrelationship with childhood asthma and allergic diseases. A large-scale cross-sectional study was performed in preschool children in Urumqi, China. Questionnaire was used to collect information on children’s health, home dampness and ventilation/cleaning (V/C) habits. Multiple logistic regressions were applied to analyze the associations between childhood asthma/allergic diseases and each sign of home dampness, dampness levels, each V/C habit and total V/C scores. The associations between dampness and health were further performed by strata analyses in two groups with low and high V/C scores. Totally 4618(81.7%) of 5650 children returned the questionnaire. Reports on home dampness were most common for water condensation on windows (20.8%) followed by damp beddings (18.0%). The most common ventilation measure was the use of exhaust fan in bathroom (59.3%), followed by daily home cleaning (48.3%), frequently putting beddings to sunshine (29.9%) and frequently opening windows in winter (8.4%). There were positive associations between the 6 signs of home dampness and children’s health particularly the symptoms last 12 months. By comparing with the reference dampness level (dampness scored 0), both the low dampness (scored 1~2) level and the high dampness level (scored 3~6) showed significantly increasing associations with childhood symptoms. There were crude negative associations between V/C habits and childhood health but not significant adjusting for home dampness levels. The risks of home dampness on children’s health were lower in the group with higher V/C score but the differences were not statistically significant. Home dampness is a potential risk factor for childhood asthma and allergic symptoms in preschool children in Urumqi, China. No significant effects were observed for ordinary home ventilation and cleaning habits in reducing the risks of home dampness on childhood asthma and allergic diseases in Urumqi, China.

## Introduction

Asthma, one of the most common chronic respiratory disorders in children, has become a severe public health problem. Although the prevalence has been reported toward stabilization or even decreasing in previously high-prevalence countries [1,2], the International Study of Asthma and Allergies in Childhood (ISAAC) concluded that, globally, there is constantly increasing prevalence of children’s asthma, rhinitis and eczema, particularly in Asia-pacific areas including China [3,4].

As a complex disease, asthma has a broad spectrum of environmental influencing factors. Over the past decades, rapid industrial and transitional economic changes have taken place in the mainland of China. The residential indoor environment has been dramatically changing. New residential buildings are constructed in a more tight style lack of good ventilation [5]. Air conditioners and/or heating/cooling systems are used more widely [6, 7] and westernized lifestyles have been more adopted in the younger generations in urban areas. Among others, lack of good ventilation and cleaning habits will further worsen the indoor air quality particularly in the presence of indoor pollution sources such as building dampness. However, few studies have reported the health impacts of both dampness and ventilation/cleaning habits in relation to childhood asthma and allergic diseases in China.

Indoor dampness has now been viewed as one potential risk factor for human health around the world, especially for children who spend a substantial fraction of time indoors [8]. Dampness is not by itself a cause for adverse health, but more as a determinant of the presence or source strength of potentially problematic exposures. It is found that dampness not only can favor house dust mites (HDM) and microbial growth, like fungi, bacteria and mold, but also can generate by itself chemical or biological degradation of building materials and furnishes. As early as in 1980’s, dampness-related problems on childhood asthma were studied in certain areas in the world [9–13], but only recently in China [14, 15].

To reduce or remove indoor dampness, there are different measures including opening windows, using exhaust fan in kitchen/bathroom, putting beddings/mattress to sunshine and home cleaning. To our knowledge, opening windows or using exhaust fan in kitchen/bathroom is helpful in reducing the indoor air humidity and increasing the fresh air supply [16, 17]. Putting beddings to sunshine, as a traditional life habit in China, helps to reduce the dampness of beddings/mattress as well as kill the biological agents including HDM in beddings/mattress [18]. Previous studies have reported the negative associations between putting beddings to sunshine and asthma or allergic diseases in Chinese children [19, 20]. Additionally, daily home cleaning by removing the surface dust or changing the beddings’ covers helps to reduce exposure to HDM allergens and microbial compounds including mould and bacteria [17]. How does the above ventilation/cleaning habits relate with the signs of home dampness and whether the good ventilation/cleaning habit is beneficial in reducing the risks of home dampness on childhood asthma and asthmatic symptoms are not clear and seldom reported in the mainland of China.

In this study, a large-scale cross-sectional survey on childhood asthma and allergic diseases in relation to home dampness and ventilation/cleaning habits was performed in children aged 1 to 8 years in Urumqi, northwest of China. With the typical temperate continental arid/semiarid climate, Urumqi's winter lasts as long as 5–6 months with severely cold climate [21]. In the last two decades, there has been more than 2-fold increase of the prevalence of asthma (from 0.4% in 1990 to 1.01% in 2010) in children aged 0–14 years in Urumqi in the longitudinal repeated nationwide survey [22, 23]. However, little is known on childhood asthma and allergic diseases in relation to home dampness as well as ventilation/cleaning habits in this area. Thus, the first aim of this study was to find out the associations between childhood asthma and allergic diseases (rhinitis ever, current wheeze, current rhinitis, current eczema, doctor-diagnosed asthma and doctor-diagnosed rhinitis) and home dampness. The second aim was to investigate the associations between childhood asthma and allergic diseases and ventilation/cleaning habits. The final aim was to explore whether the association between asthma and allergic diseases and home dampness was modified by ventilation/cleaning habits.

## Materials and Methods

### Ethics Statement

Written consents were obtained from participating parents before the study. This study was approved by the Ethic Committee of School of Public Health, Fudan University, China.

### Study Subjects

This study is part of a national epidemiological study in 10 cities in China on childhood asthma and allergies in association with home environment-China, Children, Homes and Health [24]. The current study is a cross-sectional study performed in Urumqi in November and December, 2011.

In this study, children were selected by stratified random cluster sampling in day care centers in Urumqi. Among the total 7 administrative areas in Urumqi (Kinship, Tangshan, Toutunhe, Sayibak, Midong, Shuimogou and Urumqi county), 2–4 day care centers in each administrative area were randomly selected. Parents or guardians of all children in each day care center were invited to participate in the questionnaire survey. They were asked to answer the questionnaire either at home or in schools and return the questionnaire within 3 days. Finally, 18 day care centers were included and 5650 children received the questionnaires (aged 1–8 years old). More details regarding subject selection have been published previously [20].

### Medical Questionnaire

The whole questionnaire included 4 parts of questions on children’s demographical information, childhood and parental asthma and allergic diseases, signs of home dampness and ventilation/cleaning habits.

The childhood asthma and allergic diseases were asked by using the core questionnaire in the ISAAC study [25]. They were (1) Rhinitis ever: children had ever a problem with sneezing, or a runny, or a blocked nose without a cold or the flu at any time; (2) Current wheeze: children had wheezing or whistling in the chest in the last 12 months; (3) Current rhinitis: children had sneezing, or a runny, or a blocked nose without a cold or the flu in the last 12 months; (4) Current eczema: children had eczema in the last 12 months; (5) Doctor-diagnosed asthma: children had been diagnosed as asthma by a doctor; (6) Doctor-diagnosed rhinitis: children had been diagnosed as allergic rhinitis by a doctor. The history of parental asthma/allergic diseases (PAA), defined as father’s or mother’s history of asthma, rhinitis and eczema, was also asked in the questionnaire.

### Home dampness and ventilation/cleaning habits

The information on both home dampness and ventilation/cleaning habits was collected by using questionnaire similarly in the studies in Sweden [26], Bulgaria [27], Singapore [28], USA [29] and Taiwan [30]. The questionnaire was adapted based on the local socioeconomic status, home characteristics and lifestyles in Urumqi. The information on housing characteristics including dwellings’ location (urban or suburban/rural areas) and home environmental tobacco smoke (ETS) defined as parental smoking, was also collected.

#### Signs of home dampness

Home dampness was evaluated by 6 signs of dampness: mold spots, damp stains, damp beddings, water leakage, water condensation on windows and moldy odor. For mold spots and damp stains, it was asked “Have you noticed any visible molds/damp stains on the floor, walls or ceiling in the children’s sleeping room?” (Yes vs. No); For damp beddings: “Have you noticed that your beddings or mattress are affected with dampness in the last 12 months?” (Yes vs. No); For water leakage: “Have you noticed any flooding or other kinds of water damages in the rooms in the last 12 months?” (Yes vs. No); For water condensation on windows: “Have you noticed any condensation or moisture occurred inside and at the bottom of windowpanes in the children’s sleeping room in the winter?” (>5 cm vs. ≤5 cm); For moldy odor: “Have you perceived moldy odor in your dwelling in the last 3 months?”(Never vs. Sometimes/often).

#### Dampness levels

The answer ‘yes’ to each of the 6 questions on the dampness signs was coded as 1, while the answer ‘no’ was coded as 0 (for water condensation on windows, the answer ‘>5 cm’ coded as 1 while ‘≤5 cm’ as 0; for moldy odor, the answer ‘sometimes/often’ coded as 1 while ‘never’ as 0). The dampness score for each subject was calculated by adding the coded numbers of the 6 signs of dampness (the scores ranged 0–6). Based on the dampness scores, the subjects were classified into 3 groups: the reference group with no signs of dampness (Ref_damp_, scored 0, none of the dampness signs reported), the group with a low dampness level (L_damp_, scored 1~2, 1 to 2 ‘yes’ answers out of 6) and the group with a high dampness level (H_damp_, scored 3~6, 3 or more ‘yes’ answers out of 6).

#### Ventilation/cleaning habits

Ventilation/cleaning (V/C) habits were assessed by four aspects: (1) Frequently opening windows in winter: “Do you open the windows at night when the children sleep in winter season?” (Frequently vs. Never/occasionally); (2) Frequently putting beddings to sunshine: “Do you put your beddings/mattress to sunshine in the sunny days?” (Frequently vs. Never/occasionally); (3) Daily home cleaning: “Do you clean the children’s sleeping room every day?” (Yes vs. No); (4) Bath exhaust fan: “Do you use exhaust fan in the bathroom?” (Yes vs. No).

#### Ventilation/cleaning scores

The answer of either ‘yes’ or ‘frequently’ to each of the 4 questions on V/C habits was coded as 1 while the answer ‘no’ or ‘never’ was coded as 0. The V/C score for each subject was calculated by adding the coded numbers of the 4 questions on V/C habits abovementioned (the scores ranged 0–4). Based on the V/C scores, the subjects were classified into 2 groups: the low-score V/C group (L_V/C_, referring to 0–1 positive answer out of the 4 questions) and the high-score V/C group (H_V/C_, referring to 2–4 positive answers out of the 4 questions).

### Statistical analysis

Chi-square test was used to compare the prevalence of childhood asthma and allergic diseases between groups with and without history of PPA, ETS and the dwellings’ location (urban vs. suburban/rural). The same method was used to compare the proportion of each sign of dampness between L_V/C_ and H_V/C_ groups as well as the frequency distribution of dampness levels between L_V/C_ and H_V/C_ groups. Multiple logistic regressions were applied to analyze the associations between childhood asthma and allergic diseases and the 6 signs of dampness individually, the 3 categorical dampness levels (the Ref_damp_, L_damp_ and H_damp_), the 4 ventilation/cleaning habits individually and the 2 categorical V/C levels (L_V/C_ and H_V/C_), respectively. Both the crude model (Model I) and the adjusted model (Model II) were used to analyze the associations. The crude model was set up by controlling for children’s age, gender, ethnicity, history of PAA, ETS and dwellings’ location. The adjusted model was set up by further controlling for the V/C score levels (L_V/C_ and H_V/C_) in the analyses on each dampness sign, dampness levels and childhood health, or by further controlling for dampness levels (the Ref_damp_, L_damp_ and H_damp,_ as a continuous variable) in the analyses on each V/C habit and V/C score levels and childhood health. The association trend was tested statistically between the L_damp_, H_damp_ and the Ref_damp_ level. Finally, stratified analyses in subgroup with low and high V/C scores (L_V/C_ and H_V/C_) were performed on the associations between childhood asthma and allergic diseases and dampness levels (Ref_damp_, L_damp_ and H_damp_). The interaction effect between dampness levels and V/C scores was tested. In all analyses, two-tailed tests and a 5% significant level was applied if no special statement and a 10% significant level was applied for interaction effect analysis. The association was expressed as odds ratios (ORs) with 95% confidence interval (95% CI). All statistical analyses were performed by SPSS 16.0 (Chicago, USA).

## Results

Totally, 4618 out of 5650 children (response rate 81.7%) returned the questionnaire. The average age was 4.6 ± 0.9 years (min-max 1–8 years old). Among them, 53.7% were boys and the Han nationality (China’s main nationality) people accounted for 82.4%. Most subjects lived in the urban areas (85.2%). More than half of subjects (59.2%) were exposed to home ETS and 14.2% of parents had a history of asthma/allergic diseases.

More than 40% of children reported on rhinitis ever (46.4%) or current rhinitis (42.7%) and 25.3% of children had current wheeze (Table 1). The reports on doctor-diagnosed asthma and rhinitis accounted for 8.7% and 3.7%, respectively. Children with positive reports on PAA or ETS had more reports on asthma and allergic diseases or symptoms. Children living in the urban areas reported more doctor-diagnosed rhinitis than those living in the suburban/rural areas. The detailed descriptions on childhood asthma and allergic diseases, stratified by children’s age, gender and ethnicity were described previously [20].

**Table 1:** The proportions of positive reports (%) on childhood asthma and allergic diseases/symptoms stratified by history of PAA, ETS and dwellings’ location in participants in Urumqi, China ^a^.

Symptoms/Diseases	Total	History of PAA		*P*	ETS		*P*	Dwellings’ location		*P*
	(n = 4618)	Yes	No		Yes	No		Urban	Suburban/rural	
Rhinitis ever	46.4	65.0	35.0	0.000	48.4	44.2	0.012	46.4	46.5	0.967
Current wheeze	25.3	31.4	23.5	0.000	26.9	22.7	0.003	25.4	24.8	0.744
Current rhinitis	42.7	50.9	40.8	0.000	43.9	40.5	0.031	43.1	39.9	0.145
Current eczema	5.8	9.4	5.2	0.000	6.0	5.8	0.776	6.1	4.4	0.102
Doctor-diagnosed asthma	3.5	8.9	2.6	0.000	3.4	4.0	0.326	3.7	3.3	0.616
Doctor-diagnosed rhinitis	8.7	22.8	6.2	0.000	8.7	8.6	0.866	9.1	6.4	0.028

^a^ PAA: parental asthma or allergic diseases; ETS: environmental tobacco smoke. The proportions of positive reports on childhood asthma and allergic diseases or symptoms were calculated for valid data excluding the missing values.

For home dampness (Table 2), nearly one-third of parents (27.1%) reported at least one sign of dampness in homes. For each specific sign of home dampness, the most common report was ‘water condensation on windows’ (20.8%), followed by ‘damp beddings’ (18.0%), ‘water leakage’ (15.1%), ‘damp stains’ (14.1%), ‘moldy odor’(9.1%) and ‘mold spots’(8.6%). By calculating the dampness score, it showed that 61.9% of subjects reported no signs of dampness (Ref_damp_ group, scored 0), 30.9% had a low level of dampness (L_damp_ group, scored 1–2) and 7.2% had a high level of dampness (H_damp_ group, scored 3–6). On ventilation/cleaning habits, 40.4% of subjects had 0~1 positive answer out of the 4 V/C habits and were classified into the L_v/c_ group, while 59.6% of subjects had 2~4 positive answers and were classified into the H_v/c_ group. Comparing the positive reports on each sign of dampness between L_V/C_ and H_V/C_ groups, it was found that the 6 signs of dampness were generally more reported in the L_v/c_ group. There was a statistically significant difference between the L_v/c_ and H_v/c_ group on mold spots (*P*<0.001), damp stains (*P* <0.001) and water leakage (*P* = 0.004). Comparing the frequency distribution of the 3 dampness levels (Ref_damp_, L_damp_ and H_damp_) between the Lv/c and Hv/c groups, it showed that the proportion of the H_damp_ was higher in the Lv/c group while the proportion of the L_damp_ was lower in the Hv/c group (*P* = 0.002).

**Table 2:** The proportions (%) and comparisons of each sign of dampness as well as the frequency distribution (%) of the 3 dampness levels in the total subjects and the subgroups with low (L_v/c_) and high (H_v/c_) V/C scores ^a^.

Signs of dampness and dampness levels	Positive reports	Total subjects	Subgroups ^b^		*P*
		(n = 4618)	L_v/c_ (n = 1617)	H_v/c_ (n = 2387)	
**Water condensation on windows**	>5 cm	20.8	22.8	20.0	0.076
**Damp beddings**	Yes	18.0	16.9	18.4	0.233
**Water Leakage**	Yes	15.1	17.5	13.8	0.004
**Damp Stains**	Yes	14.1	18.0	11.6	<0.001
**Moldy odor**	Sometimes/often	9.1	9.9	8.4	0.121
**Mold Spots**	Yes	8.6	11.4	7.0	<0.001
**Dampness levels**	Ref_damp_	61.9	57.2	64.1	0.002
	L_damp_	30.9	33.3	29.9	
	H_damp_	7.2	9.5	6.0	

^a^ Ref_damp_ refers to the reference group with no signs of dampness (scored 0, none of the dampness signs reported); L_damp_ refers to the group with a low dampness level (scored 1~2, 1 or 2 ‘yes’ answers out of 6) and the H_damp_ refers to the group with a high dampness level (scored 3~6, 3 or more ‘yes’ answers out of 6); L_v/c_ group refers to the low V/C score group (0–1 positive answer out of the 4 questions) and the H_v/c_ group refers to the high V/C group (2–4 positive answers out of the 4 questions).

^b^ The sum of numbers of subjects in two groups with low (L_v/c_) and high (H_v/c_) V/C scores is not necessarily equal to the total participating number due to the missing values.

There were generally positive associations between each sign of dampness and childhood asthma and allergic diseases or symptoms by multiple regression analyses in Model I and Model II (Table 3). Most associations showed stronger effects indicated by higher OR levels in model II. Among all health indicators, current asthma, current rhinitis and current eczema were more consistently associated with different signs of home dampness. In Model II, current wheeze was positively associated with all different signs of dampness with the ORs ranging from 1.39 (95% CI 1.00–1.92) for water leakage and 1.90 (95%CI 1.33–2.71) for moldy odor.

**Table 3:** Associations (OR, 95%CI) between signs of dampness and childhood asthma and allergic diseases/symptoms by multiple logistic regression analyses with (Model II) and without (Model I) additional controlling for V/C score levels ^a^.

Dampness signs	Model type	Rhinitis ever	Current wheeze	Current rhinitis	Current eczema	Doctor diagnosed asthma	Doctor diagnosed rhinitis
**Water condensation on windows**	I	**1.27(1.03,1.56)**	**1.52(1.21,1.91)**	**1.23(1.01,1.51)**	**1.62(1.09,2.40)**	1.07(0.62,1.83)	1.03(0.70,1.50)
	II	1.09(0.83,1.43)	**1.45(1.07,1.98)**	**1.31(1.00,1.72)**	1.00(0.57,1.75)	0.88(0.42,1.85)	0.86(0.51,1.45)
**Damp beddings**	I	**1.20(1.00,1.43)**	**1.59(1.31,1.93)**	**1.20(1.01,1.44)**	**1.47(1.05,2.07)**	0.96(0.59,1.57)	1.07(0.77,1.48)
	II	**1.36(1.07,1.73)**	**1.54(1.19,2.01)**	1.17(0.92,1.49)	1.48(0.98,2.27)	0.79(0.40,1.57)	1.29(0.85,1.95)
**Water Leakage**	I	**1.34(1.09,1.65)**	**1.41(1.13,1.77)**	**1.54(1.25,1.89)**	**1.93(1.35,2.76)**	1.18(0.68,2.03)	1.18(0.79,1.75)
	II	**1.39(1.04,1.87)**	**1.39(1.00,1.92)**	**1.65(1.23,2.20)**	**2.09(1.30,3.34)**	1.44(0.70,2.96)	0.87(0.49,1.54)
**Damp Stains**	I	**1.24(1.02,1.52)**	**1.57(1.27,1.95)**	**1.27(1.04,1.54)**	**1.54(1.07,2.21)**	1.26(0.76,2.08)	1.10(0.78,1.56)
	II	1.24(0.93,1.65)	**1.45(1.05,1.99)**	1.31(0.98,1.74)	**1.71(1.06,2.78)**	**1.89(1.00,3.58)**	1.02(0.61,1.71)
**Moldy odor**	I	**1.61(1.26,2.06)**	**1.91(1.48,2.47)**	**1.47(1.16,1.88)**	**1.74(1.12,2.70)**	**2.07(1.22,3.06)**	1.40(0.93,2.12)
	II	**1.59(1.14,2.22)**	**1.90(1.33,2.71)**	**1.52(1.09,2.11)**	**1.86(1.08,3.23)**	**2.46(1.24,4.89)**	1.44(0.82,2.55)
**Mold spots**	I	1.22(0.95,1.56)	**1.69(1.29,2.21)**	**1.55(1.21,1.98)**	1.46(0.91,2.33)	**2.18(1.30,3.67)**	1.21(0.80,1.85)
	II	1.35(0.93,1.96)	**1.77(1.18,2.65)**	**1.71(1.19,2.48)**	1.56(0.82,2.94)	**2.64(1.32,5.27)**	1.58(0.89,2.79)
**Dampness levels** ^b^	I	**1.29(1.10,1.52)**	**1.62(1.35,1.94)**	**1.28(1.09,1.50)**	**1.59(1.17,2.17)**	1.32(0.87,2.00)	1.20(0.90,1.62)
	II	**1.27(1.02,1.58)**	**1.59(1.23,2.06)**	**1.38(1.11,1.72)**	**1.72(1.15,2.57)**	1.44(0.87,2.39)	1.33(0.90,1.96)

^a^ Bold texts refer to the significant associations (*P*<0.05)

^b^ Dampness levels(H_damp_, L_damp_, Ref_damp_) were analyzed as a continuous variable. Model I: controlling for children’s age, gender, ethnicity, history of PAA, ETS and dwellings’ location; Model II: controlling for children’s age, gender, ethnicity, history of PAA, ETS, dwellings’ location and ‘V/C scores’.

**Fig 1:**
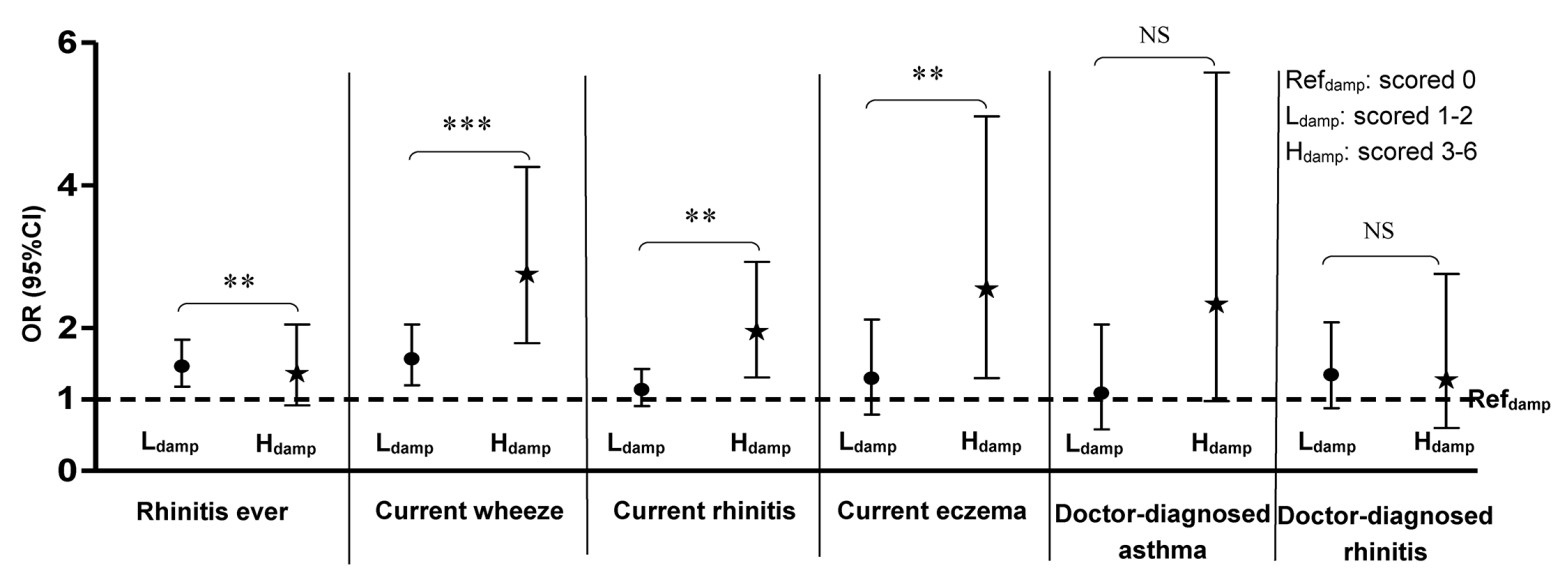
Associations (OR, 95%CI) between home dampness levels (L_damp_/H_damp_) and childhood asthma and allergic diseases controlling for V/C scores by multiple logistic regression analyses ^a^. ** *P*_*trend*_ <0.01; *** *P*_*trend*_ <0.001; NS: no statistical significance. ^a^ Controlling for children’s age, gender, ethnicity, history of PAA, ETS, dwellings’ location and V/C scores (Lv/c and Hv/c). The association trend between L_damp_ and H_damp_ in reference to Ref_damp_ was tested on its statistical significance.

In comparison with the Ref_damp_, both L_damp_ and H_damp_ levels showed positive associations with childhood asthma and allergic symptoms either as a continuous variable (Table 3) or a categorical variable in the multiple regression analyses (Fig 1). As a categorical variable, there was a significant increasing trend of positive associations from L_damp_ to H_damp_ levels in comparison with the Ref_damp_ in relation to rhinitis ever, current wheeze, current rhinitis and current eczema (all *P*_trend_<0.05).

There were generally negative associations between each V/C habit and childhood asthma and allergic diseases or symptoms in the crude model (Table 4). The negative association (OR and 95%CI) between frequently opening windows and current wheeze and current rhinitis was by 0.70(0.53–0.90) and 0.74(0.57.0.96), respectively. However, no significant association was observed between any V/C habit and childhood symptoms in Model II controlling for dampness levels. This result was the same for the V/C score level variable.

**Table 4:** Associations (OR, 95%CI) between each V/C habit, V/C scores ^a^ and childhood asthma and allergic diseases/symptoms by multiple logistic regression analyses with (Model II) and without (Model I) additional controlling for dampness levels.

Ventilation/ cleaning habits	Model type	Rhinitis ever	Current wheeze	Current rhinitis	Current eczema	Doctor-diagnosed asthma	Doctor-diagnosedrhinitis
**Frequently opening windows in winter**	I	1.23(0.95,1.60)	**0.70(0.53,0.93)**	**0.74(0.57,0.96)**	1.31(0.80,2.11)	**0.53(0.30,0.94)**	0.86(0.53,1.40)
	II	1.21(0.84,1.76)	1.53(0.99,2.35)	1.20(0.83,1.75)	1.27(0.59,2.72)	1.47(0.56,3.83)	1.60(0.82,3.10)
**Frequently putting beddings to sunshine**	I	**0.82(0.70,0.95)**	0.87(0.73,1.04)	0.97(0.83,1.12)	0.89(0.65,1.22)	**0.64(0.41,1.00)**	0.95(0.72,1.25)
	II	0.95(0.77,1.17)	0.84(0.64,1.09)	0.94(0.76,1.16)	0.76(0.47,1.24)	0.64(0.34,1.21)	1.12(0.74,1.70)
**Daily home cleaning**	I	**0.86(0.75,0.98)**	0.93(0.80,1.09)	0.91(0.79,1.04)	0.77(0.57,1.02)	0.97(0.67,1.40)	0.89(0.69,1.14)
	II	0.99(0.81,1.21)	0.92(0.72,1.17)	0.98(0.80,1.19)	1.04(0.68,1.61)	1.27(0.73,2.20)	1.04(0.71,1.53)
**Bath exhaust fan**	I	0.98(0.85,1.13)	0.92(0.78,1.08)	0.87(0.76,1.01)	1.21(0.90,1.62)	1.31(0.89,1.91)	1.24(0.96,1.61)
	II	0.91(0.74,1.12)	0.90(0.70,1.16)	0.86(0.70,1.06)	1.34(0.84,2.14)	1.73(0.94,3.20)	1.25(0.83,1.88)
**V/C scores**	I	0.96(0.83,1.11)	0.95(0.80,1.12)	**0.86(0.74,0.99)**	1.43(1.05,1.95)	1.18(0.80,1.75)	1.04(0.80,1.35)
	II	1.12(0.91,1.39)	0.96(0.75,1.24)	0.91(0.74,1.13)	1.15(0.72,1.83)	1.45(0.80,2.63)	1.39(0.91,2.11)

^a^ V/C scores have two levels-L_v/c_ (0–1 positive answer out of the 4 questions) and Hv/c (2–4 positive answers out of the 4 questions) with Lv/c as the reference level. Model I: controlling for children’s age, gender, ethnicity, history of PAA, ETS and dwellings’ location; Model II: controlling for children’s age, gender, ethnicity, history of PAA, ETS, dwellings’ location and dampness levels (H_damp_, L_damp_, Ref_damp_, as a continuous variable).

**Fig 2:**
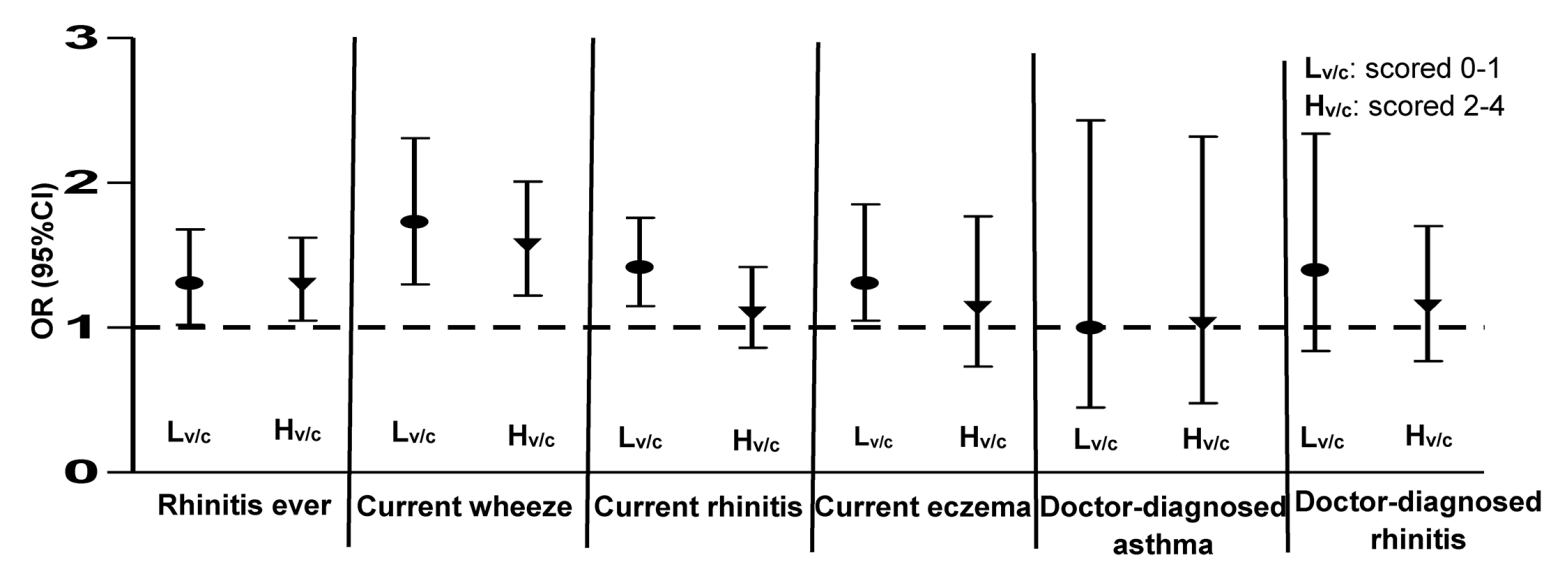
Associations (OR, 95%CI) between dampness levels (as a continuous variable) and childhood asthma and allergic diseases/symptoms in strata with L_v/c_ and H_v/c_ levels ^a^. ^a^ Controlling for children’s gender, age, ethnicity, history of PAA, ETS and dwellings’ location. The dampness levels (Ref_damp_, L_damp_ and H_damp_) were applied as continuous variable in the model.

The associations between home dampness levels (Ref_damp_, L_damp_ and H_damp_) and childhood asthma and allergic diseases or symptoms were further analyzed in two strata with Lv/c and Hv/c levels. The results showed that, there were more significantly positive associations between home dampness and childhood symptoms in the L_v/c_ group in comparison with the H_v/c_ group andthe OR levels were higher as well (Fig 2). For example, the associations (ORs and 95% CI) for current wheeze and current rhinitis in the Lv/c group were 1.73(95% CI 1.30–1.73) and 1.42(95% CI 1.15–1.76), respectively, while in the Hv/c group were 1.57(95% CI 1.22–2.01) and 1.10(95% CI 0.86–1.42), respectively. By interaction-effect test, however, the association differences between the Lv/c and Hv/c groups did not reach statistical significance (*P*_interaction_>0.1).

## Discussion

In this study, the high prevalence of childhood asthmatic or allergic symptoms in the last 12 months was identified in preschool children (1–8 yrs) in Urumqi. Home dampness was one potential risk factor for childhood asthma and allergic symptoms, while the protective effects of ordinary home ventilation/cleaning habits were not clear in Urumqi, China.

Dampness is a common phenomenon in the world. An estimated 17%-24% of homes in the Nordic countries, 37% in Canada and 50% in USA manifest signs of indoor dampness such as visible moulds on walls, floor or ceilings or water leakage [31–35]. In this study, the most common sign of dampness was water condensation on windows (20.8%), followed by other signs accounting for 8.6% -18.0%. The results mirrored that the prevalence of dampness in Urumqi was higher than that in European countries.

There are several factors related with building dampness including the poor ventilation, high humidity and the inappropriate construction design, operation, maintenance and the use of buildings [36]. Mold spots and water condensation on windows are often the signs of poor ventilation and high indoor air humidity, while water leakage and damp stains are often viewed as indicators of poor construction [19]. In this study, the high prevalence of water condensation on windows in Urumqi suggested that there was a potential lack of ventilation in these buildings. By further analyzing the relationship between dampness and V/C scores, less reports on home dampness were significantly related with higher V/C scores, particularly for opening windows in winter season, using exhaust fan in bathroom and daily home cleaning. This indicated that promoting the building ventilation/cleaning habits might benefit in reducing the home dampness in this area.

As on respiratory health in children, home dampness has been suggested as one potential risk factor for asthma and respiratory symptoms in children (e.g. wheeze and rhinitis) [36, 37]. In this study, strong positive significant associations were identified with home dampness particularly with the symptoms last 12 months, either by each individual sign of dampness or the dampness categorical levels. Despite the dampness sign indicators used in this study might be slightly different with other studies, the results were largely in agreement with most previously published studies, including cross-sectional studies [12, 27, 28, 38, 39] and birth cohort studies [40], either in China or in other countries [15, 17, 19, 20, 41–45]. Among different mechanisms which have been suggested in explaining the adverse health effects of home dampness, certain factors are mainly considered as potentially causative including the growth of bacteria, fungi, virus and HDM in indoor environment with dampness. These factors may further result in greater numbers of spores, cells, fragments, and volatile organic compounds (VOCs) emitting into indoor air [19, 46]. On the other hand, dampness itself can generate chemical or biological degradation of building materials, resulting in more emissions of VOCs such as formaldehyde [47].

Good ventilation/cleaning habits, particularly good ventilation habits were related with lower dampness in our study, suggesting that developing or improving home ventilation/cleaning habits might be beneficial in reducing the adverse effects by home dampness in Urumqi, China. Close to 60% of parents (59.3%) reported using exhaust fan in bathroom, less proportion of parents had the habits of frequently putting beddings to sunshine (29.9%) and even less on frequently opening windows in winter season (8.4%). However, opening windows as a measure of natural ventilation, together with the use of exhaust fan in bathroom as a measure of mechanical ventilation, helps to increase the interior ventilation, while putting beddings to sunshine and daily home cleaning are beneficial in reducing the microbial growth or allergen accumulation. There is evidence showing that a sufficient flow by ventilation either in natural or mechanical type is necessary to remove dampness and moisture in indoor air to reach the acceptable levels for occupants’ health [46].

In perspectives of the health effects of ventilation/cleaning habits, negative associations between ventilation/cleaning habits and childhood health were observed in crude models. However, these negative associations disappeared after controlling for home dampness levels. It suggested that in environment with dampness or potential pollution due on dampness, common ventilation or cleaning habits might have limited effects in removing the adverse health effects by dampness. There was a quasi-experimental designed study in clinically diagnosed allergy patients reported that after bedding control (including putting bedding to sunshine), there was a significant decrease of the frequency of dyspnea [18]. In this study, however, the health effects improved by ventilation/cleaning habits on childhood asthma or related symptoms were not clear.

The positive associations between dampness and childhood asthma and allergic diseases were observed in both subgroups with L_v/c_ and H_v/c_ levels, particularly for rhinitis ever and current wheeze. By comparing the ORs of these associations in the two groups, higher risks were observed in the group with L_v/c_ level but not reaching statistical significance. One recent review on respiratory tract symptoms and asthma in relation to the remediating buildings damaged by dampness and mould showed that, there was moderate-quality evidence that asthma-related symptoms and respiratory infections were improved after the intervention such as cleaning/repairing all relevant causes of mould or dampness, improving ventilation, removing damaged materials and replacing them with new ones [48]. This conclusion was mainly based on intervention studies in which the sources of dampness pollution were removed or reduced with clear targets. We did find less dampness problems in homes with higher V/C scores in this cross-sectional study. However, no sufficient evidence could be provided currently in improving the childhood respiratory health. To our knowledge, this is one of few studies focusing on both home dampness and ordinary home ventilation/cleaning habits as well as the modifying effects by ventilation/cleaning habits in this area. Further studies are needed to investigate the health effects of modifying the ventilation/cleaning status particularly in homes with dampness problems.

There are some strengths and limitations in this study. A large sample size with high response rate (81.7%) ensured the good representativeness of the aimed population at this age. No prior information was collected on participating children’ health status, dampness conditions and ventilation/cleaning habits which further reduced the sample selection bias. Parents or other guardians like grandparents of all children in the recruited kindergartens completed the questionnaire and most of parents (close to three quarters, 72.2%) lived together (in one bedroom) with their children. This indicated that the reported home dampness by parents could well reflect the dampness situation in children’s living environment. Self-reported symptoms based on questionnaire alone and lack of clinical diagnosis may cause information bias to some extent. However, the core questions on asthma and allergic diseases were the same in the ISAAC study [3, 4] which have already been validated by clinical tests in Chinese children. In summary, we do not believe that the results in this study were seriously biased by selection bias or information bias. However, the cross-sectional study in nature limited the conclusions on causal relationship which needs further investigation.

## Conclusions

In conclusion, asthma and allergic symptoms were common in preschool children in Urumqi, China. Home dampness could have adverse effects on children’s asthmatic symptoms. Developing good home ventilation/cleaning habits might benefit in reducing the dampness levels in this area. The improvement of childhood asthmatic symptoms by increasing the ordinary home ventilation/cleaning levels was not clear which needs further investigation in this area.
